# Feasibility and pilot study of the effects of microfinance on mortality and nutrition in children under five amongst the very poor in India: study protocol for a cluster randomized controlled trial

**DOI:** 10.1186/1745-6215-15-298

**Published:** 2014-07-23

**Authors:** Shalini Ojha, Lisa Szatkowski, Ranjeet Sinha, Gil Yaron, Andrew Fogarty, Stephen Allen, Sunil Choudhary, Alan R Smyth

**Affiliations:** Division of Child Health, Obstetrics and Gynaecology, University of Nottingham, Queen’s Medical Center, Derby Road, NG7 2UH Nottingham, UK; Division of Epidemiology and Public Health, University of Nottingham, Clinical Sciences, City Hospital, NG5 1 PB Nottingham, UK; Department of Community Medicine, Patna Medical College & Hospital, 800004 Patna, India; Chair of the Board of Trustees, Rojiroti UK, 32 Amenbury Lane, AL5 2DF Harpenden, UK; Pediatrics and International Health, College of Medicine, The College of Medicine, Swansea University, Room 314, SA2 8PP Swansea, UK; Secretary, CPSL, House No-22, R.L. Enclave, Duplex Colony, Near at Sonali Auto, Bye Pass Road, Vishnupuri, Anishabad, Patna, 800002 Bihar India

**Keywords:** Malnutrition, Under five mortality rate, Microfinance, Cluster randomized control trials

## Abstract

**Background:**

The United Nations Millennium Development Goals include targets for the health of children under five years old. Poor health is linked to poverty and microfinance initiatives are economic interventions that may improve health by breaking the cycle of poverty. However, there is a lack of reliable evidence to support this. In addition, microfinance schemes may have adverse effects on health, for example due to increased indebtedness. Rojiroti UK and the Centre for Promoting Sustainable Livelihood run an innovative microfinance scheme that provides microcredit via women’s self-help groups (SHGs). This pilot study, conducted in rural Bihar (India), will establish whether it is feasible to collect anthropometric and mortality data on children under five years old and to conduct a limited cluster randomized trial of the Rojiroti intervention.

**Methods/Design:**

We have designed a cluster randomized trial in which participating tolas (small communities within villages) will be randomized to either receive early (SHGs and microfinance at baseline) or late intervention (SHGs and microfinance after 18 months). Using predesigned questionnaires, demographic, and mortality data for the last year and information about participating mothers and their children will be collected and the weight, height, and mid upper arm circumference (MUAC) of children will be measured at baseline and at 18 months. The late intervention group will establish SHGs and microfinance support at this point and data collection will be repeated at 36 months.

The primary outcome measure will be the mean weight for height z-score of children under five years old in the early and late intervention tolas at 18 months. Secondary outcome measures will be the mortality rate, mean weight for age, height for age, prevalence of underweight, stunting, and wasting among children under five years of age.

**Discussion:**

Despite economic progress, marked inequalities in child health persist in India and Bihar is one of the worst affected states. There is a need to evaluate programs that may alleviate poverty and improve health. This study will help to inform the design of a definitive trial to determine if the Rojiroti scheme can improve the nutrition and survival of children under five years of age in deprived rural communities.

**Trial registration:**

Clinicaltrials.gov (study ID: NCT01845545). Registered on 24 April 2013.

## Background

The direct link between poverty and poor health was recognized in Europe in the nineteenth century [1]. More recently the World Health Organization Commission on Social Determinants of Health reported that the poor have high levels of illness and premature mortality. In all countries, at all levels of income, health and wellbeing follow a socioeconomic gradient, namely that, the lower the wealth and social class, the worse the health [2]. The United Nations Millennium Development Goals (MDGs) 1C and 4A [3] have focused the world’s attention on reducing malnutrition and mortality in children under five years of age.

Microfinance schemes provide small loans to poor families to allow them to establish income-generating activities that can potentially break the cycle of poverty. Microfinance has been shown to reduce poverty, particularly among female participants, and is recognized as a means to reduce overall village-level poverty in participating communities [4]. There is some suggestion that in addition to direct economic benefits, microfinance initiatives reduce malnutrition in children [5] and have large and positive effects on relative changes in children’s health [6]. However, there is a lack of robust evidence to support this [7] and as yet there are very few randomized control trials (RCTs) in this field of research [8].

### Need for a trial

Since its conception by Muhammud Yunus in 1978, in Bangladesh, microfinance has developed at an astounding pace. It has been credited with alleviating poverty, improving health, enhancing women’s empowerment, and bringing about a myriad other social improvements [9]. However, as early as the 1990s, the reliability of the evidence in support of microfinance was being questioned [10]. This was soon followed by the crash of the microcredit industry in Bolivia and then in several other countries, culminating in a major crisis in 2010 in Andhra Pradesh, India, where suicides among rural borrowers who faced overwhelming debts were coupled with vicious collection tactics from institutional lenders [11]. Since then there has been an increasing demand for an evidence based approach to evaluating such programs. A systematic review of the impact of microfinance on the wellbeing of poor people concluded that it could neither support nor deny the notion that microfinance is pro-poor and pro-women [8]. There is an urgent need for rigorous evaluation of microfinance programs to ensure that such initiatives indeed bring the social and health benefits that they promise and, more importantly, do not harm the communities they claim to support [12]. Randomized experiments provide a powerful method for evaluating the impact of providing microfinance to previously unserved communities [6]. Provision of microfinance is, however, a complex intervention with several social and economic interacting components. Whilst randomization may reduce bias and confounding, a careful assessment using both qualitative and quantitative methods would be required to fully evaluate the true impact of microfinance [13].

While there is a great need to resolve whether microfinance initiatives bring any health benefits to the participating communities, it is recognized that there are several potential impediments to implementing a RCT protocol and the acceptance of this approach in this setting [8]. Here we describe a pilot study to examine the feasibility of conducting a cluster randomized trial in this rural Indian community, and to make an initial assessment of the Rojiroti microfinance scheme on the nutrition and survival of children under five years of age. The results will inform the design of a larger, definitive trial of the health effects of the Rojiroti scheme.

### The Rojiroti model of microfinance

The reason cited for the disastrous consequences of conventional microfinance initiatives, such as was seen in Andhra Pradesh, is the adoption of these programs by large commercial institutions primarily motivated by profit [14]. It has therefore been suggested that microfinance providers need to return ownership to the borrowers [14]. The United Kingdom Department for International Development (DFID) supports the Rojiroti program which uses an innovative approach for providing microfinance [15]. The program is run in areas of eastern Uttar Pradesh and parts of Bihar in India by the Centre for Promoting Sustainable Livelihood (CPSL) [16]. CPSL staff enter villages and identify volunteers from the village community itself who go on to establish self-help groups (SGHs). The members of the SHG are encouraged to save money in a common pool to subsequently become eligible for a loan from the SHG itself and from CPSL. The loans are made for any purpose agreed upon within the SHG and are not restricted to conventional income-generating activities, as in conventional microfinance programs. Experience of workers in the field suggests loans are initially used for transport to government-subsidized shops and for hospital visits. As SHGs mature, later loans might be used to purchase livestock and improve housing. The approach offers a process of supporting community development at a low cost and aims to empower local villagers in managing their own finances.

### Objective

The initial objective is to determine if it is feasible and acceptable to obtain informed consent and randomize villages in Bihar to intervention or control arms of a trial, to weigh and measure children and collect mortality data. If feasible, we will also collect sufficient pilot data regarding the effects of the intervention to allow us to subsequently establish an adequately powered definitive cluster randomized trial to evaluate the effect of the Rojiroti model of microfinance on nutrition and survival of children under five years of age.

### Hypothesis

Our hypotheses are that it is feasible to conduct a cluster randomized controlled trial of the Rojiroti microfinance intervention in rural India. We also hypothesize that the introduction of Rojiroti microfinance, through establishing SHGs, will improve indicators of children’s nutrition, measured on a continuous scale, such as weight for height z-score (WHZ), weight for age z-score (WAZ), height for age z-score (HAZ), and mean mid upper arm circumference (MUAC) and that the Rojiroti microfinance intervention will decrease the prevalence of underweight, stunting, wasting, and acute moderate and severe malnutrition (as measured by MUAC) and mortality among children under five years of age.

## Methods/Design

### Study setting

**Figure 1:**
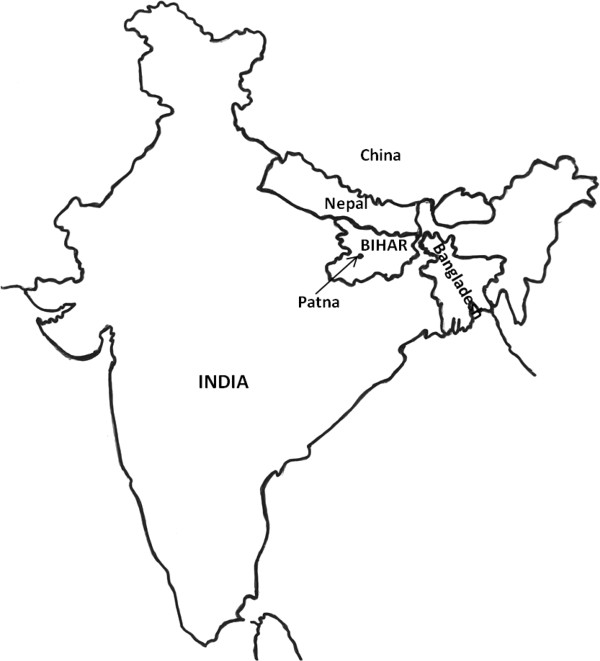
**Study site.** The study is being conducted in the Patna district in Bihar, India.

The research study is being conducted in four administrative blocks within the Patna district in Bihar: Dulhin Bazar, Naubatpur, Masaurhi, and Bikram (Figure 1). In 2011, Bihar had a population of 103,804,637, of which nearly 90% (92,075,028 people) lived in rural areas. Further demographic data about Bihar are presented in Table 1 [17]. The national poverty line used in India is based on minimum essential expenditure and is much lower than the World Bank figure – about $0.43/day [18]. Rojiroti targets women who are poor by Indian standards and hence, virtually all women who form Rojiroti SHGs will be well below the World Bank extreme poverty line. We have used the term 'very poor' to describe this group.

**Table 1:** **Demographic and health statistics on Bihar**

	India	Bihar
**Area**	3,287,263 km^2^	94,163 km^2^
**Total population (2011 census)**	1,210,193,422	103,804,637
**0-6-year-olds (2011 census)**	158,789,287	18,582,229
**Birth rate (per 1000 population in 2009)**	22.5	28.5
**Death rate (per 1000 population in 2009)**	7.3	7.0
**Infant mortality rate (per 1000 live births in 2009)**	50	52
**Sex ratio (females:males)**	940:1000	916:1000
**Literacy rate (percentage of population)**	69	62

Statistics obtained from the Statistical Year Book 2012, India [17].

### Study design

The study is a cluster RCT. The unit of randomization is the tola; a small community within a village, comprised of around 100 people. Participating tolas are all in the same agro-economic zone. Past experience of CPSL workers shows that when SHGs are set up in one tola, news spreads by word of mouth to the neighboring villages, and other SHGs are formed in the surrounding villages; to avoid such ‘viral’ spread of SHGs, the tolas in intervention arm will be at least 15 km apart from the tolas in the control arm. The process for requesting informed consent is described below. After informed consent is obtained, and prior to baseline data collection, tolas in each pair will be randomly assigned to early or late intervention.

Feasibility will be assessed in two stages. Initially, ten pairs of tolas have been selected and randomized. Baseline data will be collected from these tolas and at this point we will review the acceptability of the data collection procedures and tools and the quality of the data collected. If the feasibility of the study is established, a further 10 pairs of tolas will be similarly randomized to evaluate the effects of the intervention on nutrition status and mortality in children under five years of age.

### The intervention

CPSL staff will identify village volunteers who will steer the formation of women’s SHGs in the tolas randomized to the early intervention group. Using the Rojiroti model of microfinance [15], members of the SHG will each be asked to save a small amount (Rs. 2.50 (£0.025)) every month. When required, SHG members will be able to benefit from loans from two sources: initially from their own pooled savings and, after three to six months of running the SHG, also from CPSL. Applications for loans will be made to the SHG, and the group can apply to CPSL for the funds if it cannot provide the member with the required amount. The loans can be taken for any purpose approved by the group members. The annual interest rate on a reducing balance is 18% for all loans, providing interest payments are made and at least Rs 1 (£0.01) of the principal loan (the capital borrowed) is repaid. If Rs 1 of the borrowed amount and the monthly interest are not paid, an interest rate of 24% is charged per annum. The participant seeking the loan has to specify the loan tenure when receiving the loan; in the past CPSL have recovered between 80 and 90% of the loans within this specified time [15] and ultimately 99.3% of loans are repaid. For this study, the total duration of external credit will be recorded to analyze whether this affects health outcomes.

The tolas randomized to the control arm will not receive any intervention in the initial 18 months of the study. After 18 months, CPSL staff will enter the control tolas and provide support to establish SHGs and provide microfinance in a manner similar to the process in the tolas randomized to the intervention arm.

### Outcome measures

The primary outcome measure will be the mean weight for height z-score (WHZ) of children under five years of age in the paired early and late intervention tolas at 18 months after the scheme has been implemented in the early intervention tolas.

The secondary outcome measures will be mortality rate, overall mortality, mean weight for age z-scores (WAZ) and height for age z-scores (HAZ), the prevalence of moderate to severe underweight (WAZ < −2 SD), stunting (HAZ < −2 SD), and wasting (WHZ < −2 SD) in children under five years of age, and the prevalence of moderate to severe acute malnutrition (based on MUAC between 12.5 and 11.5 cm and MUAC <11.5 cm, respectively) in children between 6 and 60 months of age [19] in the early versus late intervention tolas 18 months from the start of the study. The same outcome measures will be analyzed at 36 months to determine if the late intervention tolas ‘catch up’.

### Determining the primary outcome measure

At the initial stages of planning the study, the primary outcome measures were mortality rate among children under five years of age and the overall mortality in the tola. However, on further literature review and discussion with experts, the primary outcome was changed to the mean WHZ of children under five years of age. Given the short duration of the study and limited number of participants, the effect of the intervention on mortality rates may not be evident. WHZ is a marker of acute malnutrition and wasting (WHZ < −2 SD) is associated with a significantly increased risk of subsequent mortality. Children with severe wasting (WHZ < −3 SD) have a 9.4-fold higher chance of dying while those with moderate wasting (WHZ −3 to −2 SD) have a 3.0-fold higher chance of dying when compared with their non-malnourished counterparts [20]. These figures are higher compared to the odds ratios for mortality for HAZ and similar to the odds ratios for WAZ. We chose WHZ as the primary outcome measure as it is strongly associated with mortality and may be affected by the intervention within the short duration of the study period.

### Qualitative measures

Qualitative data on the use of loans will be collected to examine the purpose for requesting money. The analysis will evaluate whether the purpose of loans changes in the second 18-month period of the study. Experience of CPSL workers suggests that loans are initially taken to tide over emergencies. As SHGs mature, more loans are taken for income-generating activities such as buying livestock.

### Sample size and power calculations

The data collected as part of this pilot study will be used to inform sample size calculations for a future definitive cluster RCT, using the methods described by Hayes and Bennett [21]. Published estimates of nutrition in Bihar date back to 2006 and suggest that 55% of under fives in Bihar are underweight (61% of children from scheduled castes) [22]. The pilot study will provide a current estimate of the prevalence of mortality and malnutrition in our study population.

The study will be initiated in 10 pairs of tolas to test the feasibility of recruitment, randomization, and data collection. When this is established, another 10 pairs of tola will be recruited to complete the pilot phase of the study. Based on local estimates of tola population and number of children under five years of age in each tola, 20 pairs of tolas will be sufficient to allow a power calculation to determine the sample size required to detect a statistically significant effect of Rojiroti microfinance on the primary outcome measure.

### Randomization and blinding

The 10 pairs of tolas for the feasibility phase were selected by the CPSL staff and tola names converted into code numbers which were provided to the research team based at the University of Nottingham. The code numbers were randomly assigned to either early or late intervention by the researchers in Nottingham who were blind to intervention allocation. This information was transferred back to the research team in Patna and the CPSL staff who matched the randomly assigned allocations to the tola name prior to baseline data collection. The researchers in Nottingham who will perform data analysis will remain blind to the tola allocation; however it is not possible to blind the CPSL staff and participants as the formation of SHGs and provision of microfinance cannot be masked. A further 10 pairs of tolas will be similarly randomized to complete the pilot phase of the trial.

### Data collection

Basic demographic information about each participating tola will be collected at the beginning of the study (Table 2). Information about each participating mother and her child and/or children under five-years-old will be collected using standard questionnaires. Data collected will include immunization rates among the participating children and whether they have a ‘Road to Health Card’ [23]. The Road to Health Card is a record of immunization and growth of a child. It is given to the mother when the child is born and is used to monitor child health until five years of age. Additional information about the mothers such as ability to read and write, years of schooling, freedom to travel without permission, and whether they have needed to sell assets in an emergency will also be collected. All questionnaires have been prepared in Hindi and will be communicated to the participants by researchers who can speak both Hindi and Magadhi, the local dialect of Hindi spoken in the region.

**Table 2:** **Data collected at baseline survey**

**Tola level information** (Collected from village volunteer)	How many houses are there in this tola?
	How many people live in this tola?
	How many children under 5 years of age live in this tola?
	How many children were born in the last 1 year?
	How many people have died in the last 1 year?
	How many children under 5 years of age have died in the last 1 year?
**Information about mothers** (Collected from mother)	How old are you?
	Can you read and write?
	Have you ever been to school?
	How many under children under 5 years old do you have?
	Do you or your family own any land?
	Have you or your family had to sell any assets due to emergencies such as money required for medical treatment in the last 1 year?
	Can you travel outside the village without permission from your family?
**Information about children** (Collected from mother)	Age
	Gender
	Has the child received any immunization?
	Does the child have a Road to Health card?
	Was the child born at home?

The age, weight, height, and MUAC of each child under five years of age will be recorded for all children in the tola, irrespective of whether their mothers are in the SHG. The measurement will be performed by CPSL field workers. Two workers will work together to weigh and measure each child and record the measurement that both agree on. Standing height in children aged two years and older will be measured with Seca 213 Portable Stadiometer (Seca, Birmingham, UK) to the closest 1 mm. The length of younger children will be measured using Seca 210 Measuring Mat (Seca, Birmingham, UK). Weight will be measured with Libra Fitness Standing Scale (Edryl, Goa, India); younger children will be weighed in hanging scales (Venus CHS, Ace, Rajasthan, India) and smaller infants and newborns on Docbel (Braun) Baby Scales (Popular, Docbel Industries, New Delhi, India). All weights will be recorded to the closest 100 g. MUAC will be measured and recorded to the closest 1 mm using MUAC tapes provided by the UNICEF Supply Division. CPSL staff will determine and record the number of children under five years of age who have not been measured (such as children who are temporarily absent from the village and those who are ill). This will be determined by asking the village volunteers and the mothers if they know of any children who are absent.

In addition, the village volunteer will estimate the total population, the population under five years of age, and the number of deaths in the last year (overall mortality and deaths among children under five years of age) in the tola. This information will collected using a standard questionnaire.

After 18 months, all the participating children will be reweighed and re-measured and data regarding overall mortality and mortality among children under five years of age during the study period in the tola will be collected. The CPSL-supported SHGs will then be introduced into the late intervention tolas. After 36 months from the start of the study children’s weights, heights, and MUACs will be recorded again, together with mortality data, in all tolas. At 18 and 36 months, data on deaths in each tola in the preceding 18 months will be requested from the state government (Registration of Births and Deaths) and will be used to check the validity of the mortality data obtained from questionnaires.

### Data management

Tola information and individual participant information will initially be recorded on paper questionnaires by CPSL research staff and subsequently entered into electronic data sheets by the research team in Patna and transferred to the University of Nottingham in secure password-protected files. The original data sheets will be scanned and electronic copies will be retained by the research team in Nottingham while the original papers will be retained by the team in Patna. All electronic and paper records will be filed in numerical order and stored on a secure server accessible to the research team. The electronic records will be regularly backed up the University of Nottingham.

### Evaluating the accuracy and completeness of data collection

The electronic data sheets (Excel 2007; Microsoft Corporation, Redmond WA, USA) have data validation limits set such that potentially spurious weights, heights, and MUACs will be flagged up at the time of data entry and can be rechecked against the paper records. Concordance between paper records and data entered into the electronic data sheets will be verified again by the researchers in Nottingham. A 10% random sample of all paper records will be checked against the electronic records. The Emergency Nutrition Assessment Tool [24] will be used to further check the data recorded in the electronic data sheets for spurious age, height, and weight entries such as a recorded height too small for the recorded age; any entry flagged as suspicious will also be re-checked against the paper records and any errors in data entry corrected. If any spurious data remain, we will conduct a sensitivity analysis for each of our outcome measures, excluding children with a spurious age, height and/or weight record.

### ‘Viral’ spread of self-help groups

Previous experience of CPSL in the region indicates that once SHGs begin in some tolas, information spreads by word of mouth through relatives and women in nearby tolas often set up their own SHGs (‘viral’ spread). In this trial, paired early and late intervention tolas will be at least 15 km apart in order to minimize viral SHGs being set up in the late intervention tolas. To monitor viral spread in this study, a local survey by CPSL staff will determine how often nearby tolas set up SHGs. The socioeconomic characteristics of group members in SHGs which formed virally will be compared with those of women in groups formed by CPSL. Where possible, consent for the collection of health outcome data will be taken and these data will be collected in the viral tolas similar to the data collection in the randomized tolas. CPSL staff will also record whether tolas assigned to the late intervention arm proceed regardless and set up their own SHGs during the first 18 months of the study.

### Statistical analysis

#### Type of analysis

In the feasibility and pilot study and any future trial, intention-to-treat analysis will be used, that is, where a tola has been randomized to receive microfinance early or late, their data will be included irrespective of whether the SHG and microfinance have run successfully or whether the tolas in the late intervention group formed SHGs through viral spread prior to their designated time.

#### Statistical tests

Using linear regression for continuous outcomes and logistic regression for binary outcomes, we will compare measurements from children in the early intervention tolas and children in the late intervention tolas at 18 months. We will use a multilevel model to account for the clustering of children within families within tolas, and include baseline measurements of the outcome and other confounders as model covariates. Tolas are being recruited in pairs within agro-economic zones. All tolas recruited thus far are in the same zone and all tolas in the feasibility and pilot study will be in this same zone. Given that tolas have not been matched on any other characteristics, we will not account for the pairing of tolas in our analysis.

#### Planned exploratory analyses

The feasibility and pilot phase will explore whether data can be collected on maternal literacy and years of schooling women have received. If adequate data are available, we will compare the effectiveness of the intervention according to whether the mother was literate or illiterate and whether she had received any years of schooling.

### Ethical issues and the consent process

Ethics committee approval has been granted by the University of Nottingham’s Medical School Research Ethics Committee (ethics reference number: J18102012) and by the Patna Medical College Ethics Committee (document dated 15 August 2013). Appropriate approval has also been obtained from the Department of Health, Government of Bihar, India (file number: −SHSB/NRC/2008/01/Part III/2615).

Informed consent will be obtained by a novel approach, approved by both ethics committees. We expect high levels of illiteracy among the potential participants and thus documented verbal consent may be more appropriate in this setting. The CPSL fieldworker will give a verbal explanation from an agreed script to the members of the SHG. This script has been translated into Hindi and will be explained by researchers who can speak both Hindi and Magadhi; a video of the process is available at the REACH project website [25]. In addition, information sheets are provided, translated appropriately. The project will be set up only if sufficient women (10 or more) express an interest in participating in the SHG. CPSL staff will discuss the study, explain the reasons for conducting such a study, what it would involve, and offer the option of participation. One resident of each tola will be asked to further consult with all the potential participants and give written informed consent on behalf of all the residents. Where there is no informed consent, the SHG will not have health data collected and will not be randomized to enter the study. The members of the SHGs will be requested to give verbal consent on a collective basis, using a show of hands to indicate willingness to participate. The consultation and collective consent will be video recorded. Individual parental consent for participating children will also be implied by the women volunteering to bring the children to be weighed and measured. If the women do not wish to participate in the study they will be given the option to form the SHGs and receive microfinance support outside of the trial.

## Discussion

In India, despite rapid economic growth in the past few decades and significant improvements in national average per capita gross domestic product (GDP), rates of childhood malnutrition remain among the highest in the world and progress has been slow [26]. This is demonstrated by the 12 position decline from 1995 to 2010 in India’s ranking in the Child Development Index (CDI), a global tool to assess the performance of 141 countries on child mortality, nutrition, and access to primary education [27]. Bihar remains one of the poorest states in India; in 2012 the per capita gross domestic product (GDP) in Bihar was $343 compared to the India average of $1,489 [28]. Nearly 90% of the population lives in rural areas and has inadequate access to health care and good quality education. In parallel with poverty and economic and social deprivation, Bihar has one of the worst records of children’s health and 23 districts from this state were ranked among the lowest 100 districts in the CDI and therefore included in the recent Fighting Hunger and Malnutrition (HUNGaMA) survey that focused on the nutritional status of children in some of the worst-performing regions in India [29].

In a detailed analysis of poverty and inequality in India, Rajan *et al*. [28] have suggested that to improve public health, policymakers should focus on alleviating poverty rather than simply raising average incomes, highlighting the need for novel approaches to combat poverty and malnutrition. The Rojiroti microfinance program focuses on the most deprived and very poor communities, with more than 90% of its participants belonging to the scheduled castes. These are some of the most socially disadvantaged groups and are known to suffer from inadequate access to health care which is disproportionately worse than that accessible to those of higher castes and/or better socioeconomic status [30]. This study will assess the feasibility of conducting large cluster randomized trials in some of the most deprived and densely populated communities in rural areas in Bihar [31] and will provide preliminary evidence to support or refute the use of this novel microfinance initiative in improving children’s health and nutrition.

## Trial status

The trial is currently ongoing. The first 10 pairs of tolas were randomized to early or late intervention and baseline data collection was completed in late 2013 in these communities. Further recruitment for the pilot phase is ongoing.

## Abbreviations

CDI: Child development index
CPSL: Centre for promoting sustainable livelihoods
GDP: Gross domestic product
HAZ: Height for age z-score
MUAC: Mid upper arm circumference
RCT: Randomized control trial
SHG: Self-help groups
WAZ: Weight for age z-score
WHZ: Weight for height z-score.
